# Separation of Sulfate Anion From Aqueous Solution Governed by Recognition Chemistry: A Minireview

**DOI:** 10.3389/fchem.2022.905563

**Published:** 2022-04-29

**Authors:** Si-Qi Chen, Wei Zhao, Biao Wu

**Affiliations:** Key Laboratory of Medical Molecule Science and Pharmaceutics Engineering, Ministry of Industry and Information Technology, School of Chemistry and Chemical Engineering, Beijing Institute of Technology, Beijing, China

**Keywords:** sulfate recognition, sulfate separation, liquid–liquid extraction, supramolecular chemistry, anion recognition, macrocycles

## Abstract

The sulfate anion (SO_4_^2–^) is known as an end metabolite of cysteine and methionine, and its proper concentration is associated with the expression of key functions in the physiological system. Thus, maintaining sulfate concentration at a precise level is of great significance for biology, environments, and industrial productions. Fundamental research for sulfate anion chemistry can help understand sulfate-associated physiological processes and related applications, for example, remediation. In this minireview, we summarized recent research progresses in sulfate recognition and separation using crystallization and liquid–liquid extraction. We focused on the studies wherein molecular recognition is the key element and is considered the driving force for selective sulfate separations from aqueous solution.

## Introduction

Sulfur-containing inorganic anion, mostly present as sulfate (SO_4_^2−^), is of great significance in biological, environmental, and industrial processes (Hofmeister, 1888; Markovich, 2001), for instance, sulfate is the fourth most abundant anion in human plasma and is involved in many biological processes, including biosynthesis and detoxification (Markovich, 2001). For all these processes, one of the most important factors is to control the concentration of sulfate anion. Based on the standards of drinking water from the WHO (WHO, 2017), the concentration of sulfate content in drinking water is restricted to less than 250 ppm, and excessive intake of sulfate anion may cause diarrhea. The other known example is that sulfate anion has a big impact on the vitrification process in nuclear waste treatment because of low solubility of sulfate in borosilicate glass (Moyer et al., 2012). Thus, successful technologies to separate sulfate anion are essential to maintain the concentration of sulfate anion at a proper level. Current sulfate separation techniques in industry mainly rely on precipitation (as BaSO_4_) (Benatti et al., 2009), bioreduction (Whitmire and Hamilton, 2005) or chemical reduction (Kinnunen et al., 2018), membrane technology (Wang et al., 2007), adsorption (Priyantha and Perera, 2000), and liquid–liquid extraction (Moyer et al., 2012; Ravikumar and Ghosh, 2012). However, the efficiency of sulfate separation still cannot meet the current requirement and selective separation of sulfate anion from aqueous solution remains a big challenge.

**FIGURE 1:**
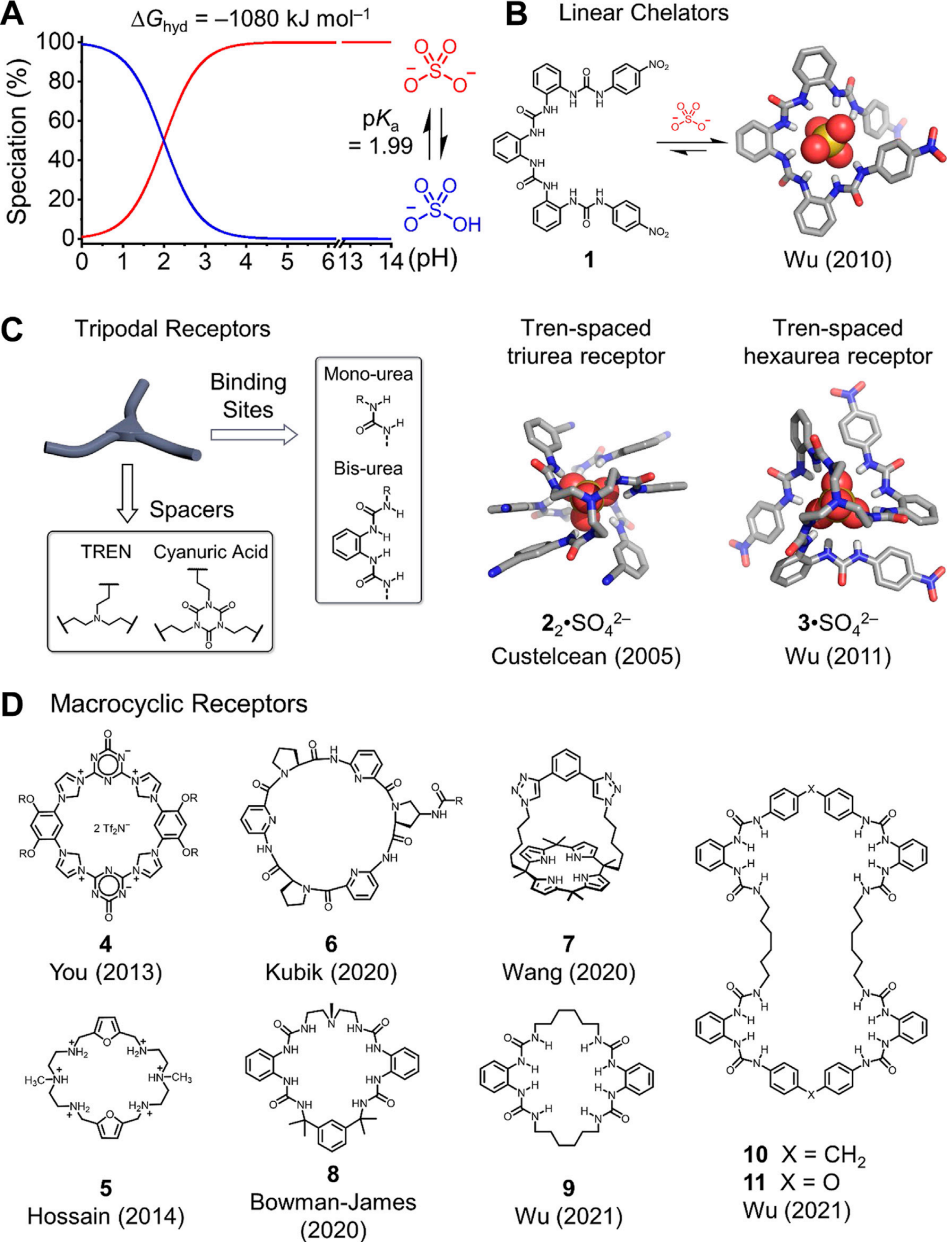
**(A)** pH-dependent nature between sulfate and bisulfate anions and representative receptors developed recently for sulfate recognition: **(B)** linear receptor, **(C)** tripodal receptors, and **(D)** macrocyclic receptors.

Recognition chemistry (Gale et al., 2016), originating from supramolecular chemistry (Lehn, 1985), offers a good way to achieve selective sulfate separation by custom-designing receptors. The programing of complementary binding sites in receptors allows for sulfate binding within well-organized binding geometry. Unlike spherical halides, sulfate is characterized by tetrahedral shape, large hydration (∆*G*_hyd_ = −1080 kJ mol^−1^), and pH-dependent speciation (mostly present as HSO_4_^−^ when pH < 1, Figure 1A). (Stern and Amis, 1959; Smith, 1977; Gao and Liu, 2004) These made the designs of sulfate-binding receptors complicated. To achieve selective sulfate separation, the designer receptors need to bind sulfate with strong affinity, thus overcoming its hydration (Yan et al., 2022). To achieve the results and inspired by the structure of sulfate-binding proteins (SBPs) in nature (Pflugrath and Quiocho, 1985; Pflugrath and Quiocho, 1988), chemists endeavor themselves to develop a variety of receptors with programed hydrogen-binding sites for sulfate recognition. Previous studies before 2011 have been well-summarized by Ghosh Ravikumar and Ghosh (2012) and Moyer Moyer et al.(2012). In this *minireview*, we focus on the progresses made in the last decade and highlight the representative studies for sulfate separation from water using crystallization and liquid–liquid extraction (LLE). Notably, we also comment on the correlations between sulfate recognition and separation, which would help us understand design principles of synthetic receptors for selective sulfate separation governed by recognition chemistry.

## Sulfate Recognition Using Synthetic Receptors

Anion coordination chemistry (Dietrich et al., 1984), known as anion receptor chemistry, was proposed by Lehn Lehn (1978) and further defined and elucidated by Bowman-James Bowman-James(2005). Most studies of anion receptor chemistry focus on developing receptors for commonly seen anions (Gale et al., 2016), for example*.*, halides and nitrates. In contrast, studies on sulfate anions and other oxyanions are largely unexplored, which are restricted by their relatively large hydration and pH-dependent speciation (Figure 1A). In early examples, receptors used for bisulfate anion (HSO_4_^−^) binding are widely developed because of bisulfate’s relatively less hydration energy (Fatila et al., 2016; Fatila et al., 2017). However, the bisulfate anion only presents in acidic solution (pH ≤ 4), thus limiting related applications. Up to date, various sulfate-binding receptors have been designed and synthesized (Ravikumar and Ghosh, 2012). Among recently reported studies, the designed receptors can be classified into three types based on their structural geometries: linear chelators, tripodal receptors, and macrocyclic receptors.

Linear chelators and tripodal receptors are first used for sulfate recognition through hydrogen bonding or electrostatic interactions that are deliberately programed into the main backbone of receptors. Typical electrostatic binding sites are ammonium and guanidinium, hydrogen-binding moieties which include amine, amide, and urea (Gale et al., 2016). Given the large dipole moment (∼4.1 D) and rigidity of the urea unit, Wu et al. developed *ortho*-phenylene spaced tetra-urea receptor **1** (Figure 1B) that can fold in helical conformation when binding to sulfate through eight hydrogen bonds, based on an X-ray crystal structure (Wu et al., 2008; Jia et al., 2010). Nuclear magnetic resonance (NMR) titration suggests that the tetra-urea receptor **1** binds to sulfate anions with > 10^4^ M^−1^ in 10% (v/v) D_2_O with DMSO-*d*_6_, and the binding affinity for the naphthyl-substituted version of receptor **1** is suggested to be 10^6.27^ M^−1^ based on fluorescent spectroscopic titration. Other oligoura-based linear chelators with selective sulfate bindings are also developed by Bowman-James (Jia et al., 2015).

Compared to linear chelators, tripodal receptors provide extra arms for sulfate binding with better complementarity and stronger binding affinity. The studies on these tripodal tris-urea and hexa-urea receptors have been documented in the previous review (Moyer et al., 2012; Ravikumar and Ghosh, 2012). Representative tripodal receptors mostly consisting of a *C*_3_ symmetric bridging spacer and hydrogen binding site, mono-urea, and *ortho*-phenylene bis-urea are typically used (Figure 1C). For the *C*_3_ symmetric spacer, tris (2-aminoethyl)amine (TREN) (Ravikumar and Ghosh, 2012) and cyanuric acid are commonly utilized (Dutta and Ghosh, 2013; Dutta et al., 2015). According to the principle of anion coordination chemistry, the coordination number for sulfate anion is 12 (Bowman-James, 2005) because sulfate consists of four oxygen atoms that can accommodate up to 12 hydrogen bonds to satisfy the binding geometry. In particular, in the cases of tripodal tris-urea receptors, two receptors are essential to bind one sulfate anion by forming a 2:1 sandwich complex. In contrast, a single hexa-urea receptor is sufficient to bind one sulfate anion.

A pioneer work from Custelcean reported a 2:1 receptor-to-sulfate sandwich complex using a TREN-based tris-urea receptor with a terminal cyano-substituted phenyl ring (Custelcean et al., 2005). This 2:1 complex shows the highest coordination number (12 hydrogen bonds) of sulfate anion, which can also be achieved using the hexa-urea receptor (Figure 1C). The hexa-urea receptor developed by Wu et al. provides exceptional and complementary tetrahedral space for sulfate encapsulation (Jia et al., 2011). Compared to tris-urea receptors, the hexa-urea receptors are indicated to bind sulfate stronger because of the favorable entropic contribution from pre-organized conformation. The X-ray crystal structure confirms the sulfate binding through 12 hydrogen bonding. ^1^H NMR titration suggests a strong binding constant of over 10^4^ M^−1^ in 25% (v/v) D_2_O with DMSO-*d*_6_. Hossain et al. found that the hexa-urea receptors bearing the *meta*-nitrophenyl group or pentafluorophenyl group bind to sulfate in DMSO with binding affinities of 10^5.78^ M^−1^ and 10^5.55^ M^−1^, respectively (Portis et al., 2017). By changing the TREN spacer to a relatively rigid cyanuric acid spacer, the corresponding tris-urea and hexa-urea receptors display comparable binding properties for sulfate.

Transition from linear and tripodal receptors to macrocyclic shape benefits from the reduced entropy cost for the pre-organization of receptors with enhanced sulfate binding affinity (Dietrich et al., 1984). Electrostatics offers a stronger contact than hydrogen bonds and is widely programed into the macrocyclic backbones for sulfate binding. In 2013, You et al. designed a highly rigid tetrakisimidazolium macrocycle **4** with two positive charges (Zhou et al., 2013). This receptor shows selective turn-on fluorescence upon sulfate binding with an exceptionally strong binding affinity of 8.6 × 10^9^ M^−2^ in water. The X-ray crystal structure shows that one sulfate anion is stabilized by two macrocycles through electrostatics, hydrogen binding, and π–π interactions. The hexaazamacrocyclic receptor **5** with four positive charges, developed by Hossain et al., also displays selective sulfate binding in water (Rhaman et al., 2014). A 1:1 complex is seen in crystal, and the binding affinity is determined to be 10^4.43^ M^−1^ in water.

Neutral macrocycles consisting of hydrogen bond donors have also been developed for sulfate recognition. Kubik et al. developed a series of cyclopeptide-based macrocycles showing selective anion binding in an aqueous medium (Kubik, 2010). By cooperating gold nanoparticles with the cyclopeptide **6**, they observed selective sulfate sensing (co-precipitation of sulfate-bound nanoparticles) in water (Bartl et al., 2020). The cyclopeptide **6** is suggested to form a 2:1 sandwich complex similar to that for tetrakisimidazolium macrocycle **4**. Calix [4]pyrrole is a classic macrocycle for anion recognition; Sessler, Moyer, and co-workers have developed a family of calix [4]pyrrole–based macrocycles for anion recognition and separation (Eller et al., 2007; Moyer et al., 2010; Borman et al., 2011). Very recently, Wang designed a calix [4]pyrrole strapped benzenebistriazole bis-cycle **7** that displays strong sulfate-binding affinity of > 10^6^ M^−1^ in an aqueous medium (He et al., 2020). According to the X-ray crystal structure, this bis-cycle binds to the sulfate anion in a 1:1 stoichiometry, stabilizing by multiple N–H and C–H hydrogen bonds.

Other recently developed macrocyclic receptors use urea units as the hydrogen-binding sites for sulfate recognition (Kaur et al., 2020; Zhao et al., 2021). In 2020, Bowman-James et al. developed a semirigid tetra-urea macrocycle **8** displaying 1:1 sulfate binding based on the X-ray crystal structure (Kaur et al., 2020). The binding affinity is determined to be 9.0 × 10^4^ M^−1^ according to ^1^H NMR titration in 0.5% (v/v) D_2_O with DMSO-*d*_6_. Very recently, we developed a family of tetra-urea– and octa-urea–based macrocycles that can be readily prepared using a modular, two-step strategy from commercially available building blocks (Zhao et al., 2021). The monomer sequences for these macrocycles rely on the flexibility of chosen spacers. In particular, tetra-urea macrocycles are selectively formed using rigid diphenyl methylene and diphenyl ether spacers, which show interesting sulfate-binding channels in solid state. The sulfate anions bind to macrocycles and water molecules through hydrogen bonding for the formation of 1D sulfate channels. A relatively flexible tetra-urea macrocycle **9** can also be prepared using a bottom-up strategy and shows full encapsulation of the sulfate anion in the central cavity. By comparison, octa-urea macrocycles **10** and **11** are prepared using both relatively rigid and flexible spacers. Notably, these two octa-urea macrocycles display unusual encapsulation of two sulfate anions with significantly different binding geometries. The octa-urea macrocycle **10** binds sulfate with a “figure-eight” conformation, and the other octa-urea macrocycle **11** forms a mesocate conformation.

The use of molecular receptors provides an effective and achievable way for selective sulfate binding that can be further utilized for sulfate separation. To separate sulfate anion from water, synthetic receptors with strong sulfate binding affinity and selectivity are essential. In the following sections, we will discuss the progresses made recently for sulfate separation that are based on recognition chemistry (Moyer et al., 2012). Here, we focus on the methods of crystallization and liquid–liquid extraction.

## Sulfate Separation Using Crystallization

**FIGURE 2:**
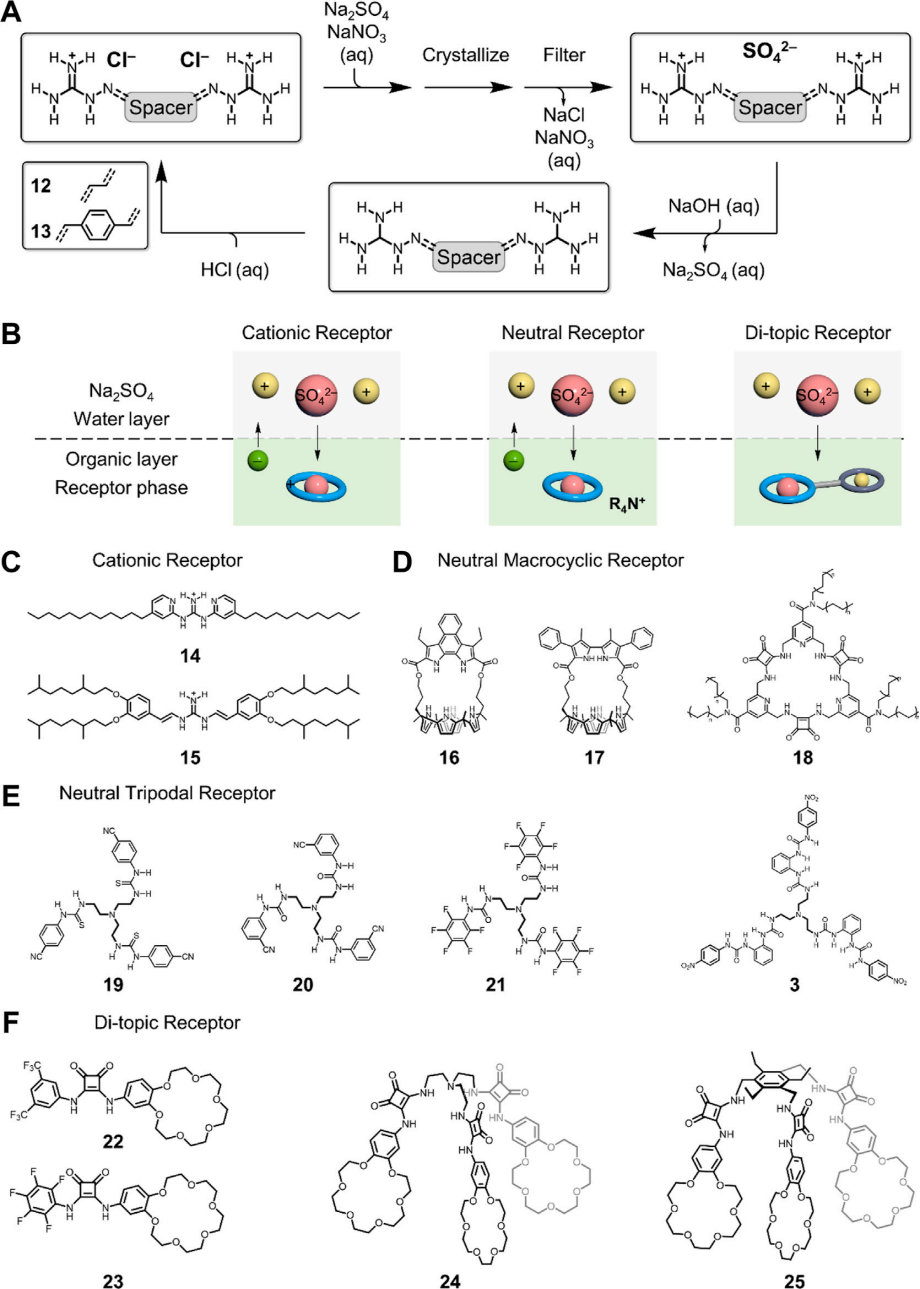
Sulfate separation using **(A)** crystallization and **(B)** liquid–liquid extraction. Representative receptors used in liquid–liquid extraction: **(C)** cationic receptors, **(D)** macrocyclic receptors, **(E)** tripodal receptors, and **(F)** di-topic receptors. It is to be noted that the cyclic shape of the receptor seen in figure (2b) does not represent the actual shape for receptors used for sulfate separation.

The crystallization of sulfate anion was performed using water-soluble receptors to bind sulfate and form water-insoluble complexes, thus precipitating out (Custelcean et al., 2008; Custelcean and Remy, 2009; Custelcean et al., 2010; Rajbanshi et al., 2011; Rajbanshi and Custelcean, 2012; Custelcean et al., 2015a). The receptors utilized for crystallization need to bind the sulfate anion with strong binding affinity, and the formed sulfate complexes should be able to build contacts with neighboring complexes, thus forming aggregates. The key element is that the formed aggregates arrange with well-defined arrays in the solid state, which is not soluble in a given aqueous solution. Pioneer works from Custelcean and others developed a series of tripodal tris-urea receptors functionalized with terminal pyridyl groups showing selection sulfate separation from aqueous alkaline solutions (Wu et al., 2008; Custelcean et al., 2005; Custelcean et al., 2008; Custelcean and Remy, 2009; Custelcean et al., 2010; Rajbanshi et al., 2011; Rajbanshi and Custelcean, 2012; Custelcean et al., 2015a). In crystal structure, it is the terminal pyridyl groups that allow connecting the discrete complexes through metal coordination or hydrogen bonding. Alternatively, by using bis(guanidinium)-based linear receptors, **12** and **13** (Custelcean et al., 2015b; Custelcean et al., 2016), the sulfate anions can also be separated from the nitrate-rich solution by crystallization (Figure 2A). The X-ray crystal structures suggest that sulfate anions are clustered with water molecules and co-stabilized by receptors through electrostatics and hydrogen bonding. The receptors can be recovered by being treated with sodium hydroxide solution and consequently acidified with hydrochloric acid for the next cycle. The receptor-assisted crystallization of sulfate is one of the most effective techniques for sulfate separation. Compared to precipitation of inorganic salts (BaSO_4_) (Benatti et al., 2009), receptor-assisted crystallization shows better selectivity. However, the current methods usually take more time (days) to complete the full process of crystallization, which may limit its application in the industry.

## Sulfate Separation Using Liquid–Liquid Extraction

Liquid–liquid extraction is another widely used technique for sulfate separation that can be readily integrated with actual infrastructures in the industry (Moyer et al., 2012). Compared to crystallization, LLE is more dependent upon receptor designs, yet requires less operating time for all-liquid handling. The desirable receptor (or extractant) needs to be soluble in a water-immiscible solvent, hydrophobic, and binds sulfate with strong affinity in a selective manner. The transport of sulfate from water into the other immiscible solvent can be defined as the competition between the hydration and binding affinity, which is akin to the design principles for the crystallization process. The difference is that LLE can complete in seconds, but the overall charges across two layers need to be leveraged either by the receptor or extra reagents, for example, ammonium (Borman et al., 2011). Based on the receptors adapted for LLE, there are three major types: cationic or di-topic receptors without phase transfer–assisted reagents and neutral receptor with phase transfer–assisted reagents (Figure 2B). In particular, for cationic receptors, their corresponding counter-anions (mostly Cl^−^ or NO_3_^−^) can move into water for charge neutrality. Similarly, the counter-anions of ammonium salts (R_4_N^+^) are used when using neutral receptors (Kim et al., 2014). In the case of di-topic receptors, both sulfate anion and the corresponding cations (Na^+^ or K^+^) can be extracted into the organic layer simultaneously. In all these cases, recognition of sulfate anions by the designer receptor is the driving force for extraction.

Moyer et al. have made significant contributions to the field of sulfate separation, especially in separating sulfate from nuclear waste (Moyer et al., 2012). Recently, they found that the use of simple guanidinium-based receptors **14** and **15** can also extract sulfate anion from water (Seipp et al., 2018; Williams et al., 2018). Electrostatics between positively charged guanidinium and sulfate anion in a 1:2 stoichiometry is the driving force for binding and separation. By comparison, better selectivity of sulfate separation is observed for receptor **15** owing to the formation of reverse-micelles. In addition, **15** is of better synthetic feasibility, higher sulfate separation efficiency, and process compatibility for industrial use. In these studies, counter-anions of guanidinium receptors move into the water layer for charge neutrality.

As an alternative to cationic receptors, macrocyclic receptors with pre-organized conformations can also be used for sulfate separations. Recently, Moyer, Sessler, and co-workers developed cage-type bipyrrole-strapped calix [4]pyrroles **16** and **17** showing selective sulfate extraction from water (Kim et al., 2014). Unlike cationic receptors, a phase transfer reagent is required for neutral receptors to leverage the overall charges across two layers. In their studies, the commercially available methyltrialky (C_8-10_)ammonium (A336^+^) as a chloride salt was used. Taking the design principles for sulfate recognition, pro-organized conformations for macrocyclic receptors can save the entropic cost, thus retaining strong binding affinity and efficient sulfate extraction. The complexed sulfate structures are demonstrated by X-ray diffraction analysis, and the well-defined 3D cage-type conformation enables strong sulfate binding affinity and selective extraction.

Another typical macrocyclic receptor recently developed for sulfate extraction is macrocyclic squaramide (Qin et al., 2016; Qin et al., 2019; Qin et al., 2020). Given the high dipole moment (∼7.4 D) of the squaramide unit, Jolliffe et al. developed a series of squaramide-based macrocycles (Qin et al., 2016; Qin et al., 2019; Qin et al., 2020). The *meta*-phenylene–spaced tris-squaramide macrocyclic receptor is demonstrated to selectively bind sulfate with over 10^3^ M^−1^ in a highly competitive aqueous solution of 1:1 v/v H_2_O:DMSO (Qin et al., 2016). By changing the phenyl spacer to the pyridyl version, the corresponding receptor was found to retain the selective sulfate binding across a wide pH range (3.2‒14.1) (Qin et al., 2019). With further modification of using the aliphatic chain, the designer receptor **18** can extract sulfate from water (pH = 3.2‒9.4) into a chloroform phase (Qin et al., 2020). The protonation of the isonicotinamide unit in the receptor is proposed to offer extra electrostatics for sulfate binding. Interestingly, the Cram U-tube experiment suggests that the dynamic transport of sulfate can be realized through the chloroform liquid membrane from the source phase to the receiving phase (BaCl_2_ solution). The sulfate anion is precipitated out as BaSO_4,_ and the receptor is recycled for next use.

In addition to the aforementioned characteristic sulfate-binding properties, tripodal receptors are suggested to be good extracts for sulfate separation. Ghosh et al. developed a series of tris-thiourea– and tris-urea–based receptors, **19**, **20,** and **21**, showing selective sulfate extraction (Dutta and Ghosh, 2013; Dutta et al., 2014; Dutta et al., 2015). The tripodal hexa-urea receptor **3** is demonstrated to be able to extract sulfate from water into chloroform (Jia et al., 2011). For these studies, phase transfer reagents are essential for two reasons: maintaining charge neutrality and improving the solubility of un-complexed receptors that are typically insoluble in less polar solvents. To avoid the use of phase transfer reagents and maintain charge neutrality, the di-topic receptor that can simultaneously bind anion and the counter-cation is ideal.

The di-topic receptor comprises anion-binding sites and cation-binding sites, which is well-known in anion recognition chemistry yet rarely used for sulfate recognition and exaction. Recently, Romański et al. developed a new family of di-topic receptors based on squaramide and crown ether moieties for sulfate extraction from alkaline solutions (Figure 2F). (Jagleniec et al., 2019; Zaleskaya et al., 2020a; Zaleskaya et al., 2020b; Jagleniec et al., 2021) The linear di-topic receptors **22** and **23** are demonstrated to bind sulfate in a 4:1 receptor: sulfate stoichiometry as suggested by X-ray crystal structures (Jagleniec et al., 2019; Zaleskaya et al., 2020b). Akin to the structure of SBP, four squaramide units combine together to support binding with one sulfate anion through eight hydrogen bonding. The transport of sulfate anions in a U-tube has also demonstrated and indicated that 31% of sulfate is delivered in 14 days. Evolving from linear di-topic receptors, tripodal versions **24** and **25** were also developed and suggested to extract sulfate with 49 and 72% efficiency, respectively (Zaleskaya et al., 2020a; Jagleniec et al., 2021). These studies are the first examples of separating the sulfate anion as alkaline salts from water, which open the opportunity to develop new sulfate separation receptors that do not need to rely on phase transfer reagents in liquid–liquid extraction.

## Conclusion and Future Outlook

In this *minireview*, we summarized recent studies for sulfate recognition and separation using crystallization and liquid–liquid extraction. We observe that significant progresses have been made in developing synthetic receptors for sulfate recognition, which are thus utilized for sulfate separation. The fundamental challenge is still in understanding the design principles of the receptor with strong binding affinity and selectivity for sulfate. More studies on receptor designs for sulfate recognition and separation are needed that can help accumulate sufficient examples for understanding of receptor designs. Future studies include but are not limited *1*) to design receptors with size-complementary geometry and characteristic hydrogen-bonding donors for sulfate binding, for example, *ortho*-phenylene spacer bis(urea) (Zhao et al., 2021), squaramide (Qin et al., 2016), and directional halogen bond (Pancholi and Beer, 2020); *2*) to design new receptors for sulfate recognition in pure water (Langton et al., 2016); *3*) to design new receptors with strong sulfate binding affinity, for example, bicyclic cage-type receptor (Liu et al., 2019); *4*) to design new receptors for efficient sulfate extraction (Dietrich et al., 1984); *5*) to understand the dynamic process in liquid–liquid extraction, for example, phase–phase transfer and equilibria across multilayers (Moyer et al., 2012); *6*) to understand the correlation of recognition and separation, for example, correlation of binding affinity and extraction efficiency, and *7*) to develop new sulfate-related application, for example, direct absorption of SO_x_-containing gas by receptor solutions (Martínez-Ahumada et al., 2021).
